# Incidence of acute kidney injury among COVID-19 patients in Egypt

**DOI:** 10.1186/s41100-021-00356-6

**Published:** 2021-06-14

**Authors:** Enass E. El-Sayed, Abdou K. Allayeh, Amany A. Salem, Sheren M. Omar, Salwa M. Zaghlol, Hala M. Abd-Elmaguid, Mohammed M. Abdul-Ghaffar, Magdy M. ElSharkawy

**Affiliations:** 1 Department of Nephrology, Ahmad Maher Teaching Hospital, Cairo, Egypt; 2 Virology Laboratory 167, National Research Centre, 33 El-Buhouth Street, Dokki, Virology Lab 176, Water Pollution Research Department, National Research Centre, Cairo, Egypt; 3 Faculty of Medicine, Cairo University, Cairo, Egypt; 4 Department of Gastroenterology, Ahmad Maher Teaching Hospital, Cairo, Egypt; 5 Damanhour National Institute, Damanhour, Egypt

**Keywords:** Acute kidney injuring, COVID-19, Incidence, Mortality, Hemodialysis

## Abstract

**Background:**

Despite the fact that the fundamental characteristics of coronavirus disease-2019 (COVID-19) are respiratory manifestations, multi-organ failure including the kidney has been documented. There are no clear comparisons of COVID-19 cases with and without acute kidney injury (AKI) to show whether there are aspects of acute kidney injury progression path or outcome that are unique to this disease.

**Methods:**

In this work, we analyzed the data of 734 COVID-19 cases admitted to the Ahmad Maher Teaching Hospital in Cairo, Egypt, between June 6 and July 25, 2020. Data on demographics, comorbidities, laboratory results, and outcomes were assessed. To assess the incidence rate of AKI in Egyptian COVID-19 patients, comparisons were carried out between home-isolated COVID-19 patients, hospitalized COVID-19 patients, and ICU COVID-19-patients with or without AKI.

**Results:**

AKI was more common in hospitalized mild COVID-19 patients than in home-isolated and ICU COVID-19 patients (15.0% versus 10.8% and 14.2%, respectively). The overall occurrence rate of AKI was significantly higher in COVID-19 patients (n=91, 14%). Hemodialysis, on the other hand, was required in 76% of the extreme ICU COVID-19 patients who developed AKI (22/29). The absolute number of patients with AKI COVID-19 who required hemodialysis was 34 (37%). This accounted for 5.2% of all COVID-19 patients and 37% of those with AKI. The mortality rate in COVID-19 patients with or without AKI was 15.4% and 4.8%, respectively.

**Conclusion:**

AKI in our COVID-19 patients is associated with a high mortality rate in ICU-COVID-19 patients. Our findings suggest that COVID-19 patients, particularly ICU COVID-19 patients, should be closely monitored for the development of AKI. Early identification of AKI, as well as prompt intervention, can improve COVID-19 patient outcomes.

## Introduction

COVID-19, a novel respiratory illness caused by SARS-CoV-2, was discovered for the first time in Wuhan in December 2019. COVID-19 symptoms range from a common cold to a high fever and acute respiratory distress syndrome (ARDS), which causes multi-organ failure such as kidney disease [1]. In COVID-19 cases, the kidney may be a target for organ injury due to angiotensin converting enzyme-2 (ACE-2), the coupling site for SARS-CoV-2 that is highly expressed in proximal tubule cells and podocytes. AKI has been identified as an extreme COVID-19 obstruction with a higher risk of death in critically ill patients based on several case series and retrospective reports [2, 3]. In early studies conducted in China and Italy, the frequency of AKI varied widely from 0.5 to 29%. Data from the USA were restricted ICU patients with a high incidence rate of 37 to 40% and an in-hospital mortality rate of 35 to 41% [2]. The AKI incidence is generally high in some reports and negligible in others. For instance, Guan et al. [1] found that the AKI frequency was only 0.5% in a multicenter study of 1019 cases. However, Cheng et al. [4] found that in a single center study the AKI prevalence was 3.2%. Two distinct cohorts revealed a significantly higher occurrence of AKI. The first cohort had 66% AKI rate in a cohort of 193 serious patients, while the second cohort had 50% AKI rate in non-survivors [5–7]. So far, little information on AKI in COVID-19 has been published in Egypt. The goal of this study is to assess the occurrence of AKI in Egyptian COVID-19 patients as well as the clinical features and outcomes associated with AKI in COVID-19 patients.

## Subjects and methods

### Study design

In this cohort study carried out in a single hospital serving a low-income community in Cairo, Egypt, all individuals greater than 18 years old admitted to the Ahmad Maher Teaching Hospital with COVID-19 between June 6 and July 25, 2020, were examined. This hospital is one of the few hospitals to have the largest number of COVID-19 patients admitted. Infection has been diagnosed dependent on clinical manifestations, lung irregularities, and a positive consequence of real-time PCR for nasal or pharyngeal specimens. We eliminated COVID-19 patients from our study if they were undergoing maintenance hemodialysis or were renal transplant recipients. The ethical committee of the General Organization of Teaching Hospitals and Institutes in Egypt has endorsed this research (approved number HAM00125). We did not need patient consent, due to its retrospective nature.

### Data collection

From the medical record, we gathered demographics, clinical symptoms, clinical history, comorbidities, and other laboratory information. Complete blood picture, kidney and liver functions, and inflammatory markers were the lab data. The lab offered the standard scope of these tests. We referred to Enhancing Global Outcomes definition for the analysis of AKI, which utilized the peak serum creatinine incentive to assess the AKI, utilizing an increase of 0.3 mg/dl or half an increment in serum creatinine from the standard creatinine to greatest creatinine in-clinic [8]. AKI occurrence is an essential outcome, while the other outcomes include hemodialysis treatment and hospital death rate.

### Definitions

All patients in the present study were classified into three groups based on specific criteria as the next: (1) home-isolated COVID-19 patient group includes all patients who had COVID-19 pneumonia diagnosed by a typical chest computed tomography and a positive PCR of a nasopharyngeal sample for SARS-CoV-2 with mild symptoms and did not need for supplementary oxygen. (2) Hospitalized COVID-19 patient group includes all patients who had COVID-19 pneumonia diagnosed by a typical chest computed tomography and a positive PCR of a nasopharyngeal sample for SARS-CoV-2 with requirement for supplementary oxygen, furthermore, met any of the following criteria: (A) respiratory distress, defined as the respiratory rate ≥30 times/min, with cyanosis; (B) arterial digital oxygen saturation ≤93% (at room air); (C) the ratio of partial pressure of oxygen to the fraction of inspired oxygen (PaO_2_/FiO_2_) ≤300 mmHg (1 mmHg = 0.133 kPa). (3) ICU COVID-19 patient group includes all patients of hospitalized group admitted to ICU stay and met any of the following criteria: (1) respiratory failure that requires mechanical ventilation; (2) shock; (3) multiple organ failure that requires ICU life support.

The Acute Disease Quality Initiative 16 consensus group defines AKI recovery as the absence of AKI by both serum creatinine and urine output criteria (per KDIGO) within 7 days of AKI onset. When compared to persistent AKI, transient AKI is defined by rapid reversal of AKI within 48 h and is strongly associated with lower morbidity and mortality, which is defined by reversal of AKI within 2–7 days. AKI that does not recover within a week is termed acute kidney disease (AKD; a term first proposed in the original KDIGO AKI guidelines); AKD is assigned stages 1–3 on the basis of the occurrence of KDIGO AKI criteria during the 7–90-day period after the initial AKI. Patients in stage 0 subgroups A–C do not meet the criteria for stages 1–3, but they are at a higher risk of developing CKD (lesser decrements of GFR, loss of kidney functional reserve, proteinuria, and other markers of kidney damage). Finally, chronic kidney disease (CKD) is defined as kidney disease that lasts 3 months or longer [9].

### Statistical analysis

Continuous variables have been described as mean ± standard deviation and interquartile range. As categorical variables, frequency and percentage were presented. The mean of continuous variables was compared using independent *t* test. Odds ratios (ORs) with 95% confidence intervals (95% CI) were obtained to assess the statistical significance using the MedCalc Software Version 18.

## Results

**Fig. 1:**
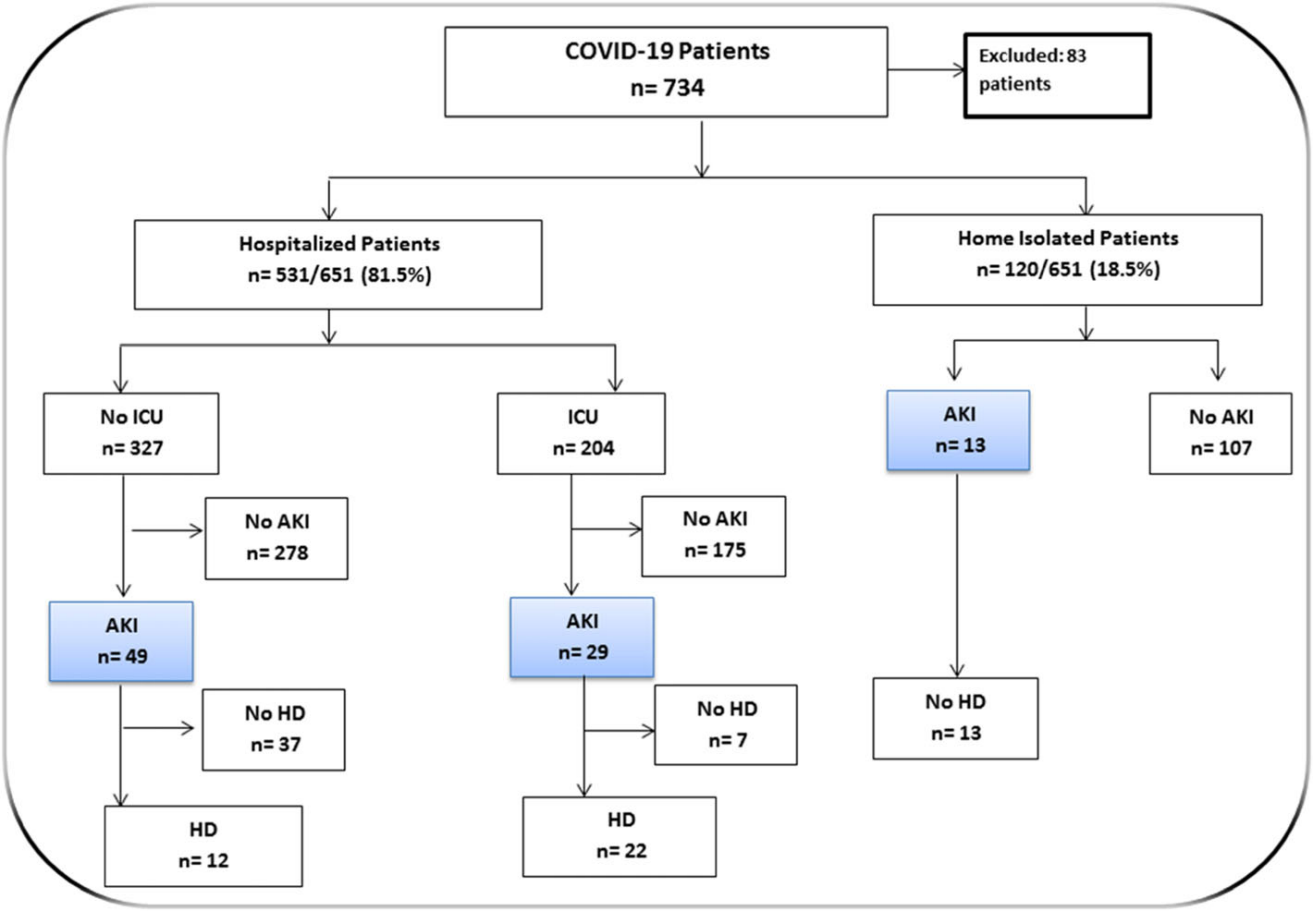
Flowchart of the incidence of AKI in Egyptian COVID-19 patients. AKI, acute kidney injury; HD, hemodialysis

A total 734 COVID-19 patients were admitted to the Ahmad Maher Teaching Hospital in Cairo, Egypt, during the period from 6 June to 25 July, 2020. We excluded 83 patients admitted on different levels of maintenance dialysis and recipients of renal transplant. The data of 651 COVID-19 patients were included in the comprehensive analysis in this study. The overall incidence of AKI among COVID-19 patients was 14% (91/651); AKI developed in 78 hospitalized COVID-19 mild/severe patients (14.7%) and 13 home-isolated COVID-19 patients (10.8%) (Fig. 1). The clinical features of patients with COVID-19 are described in Table 1. The median age was 56 years, with 57.9% of patients being male (377/651). The characteristics of COVID-19 patients without AKI are presented with cough (91.4%), fever (54.3%), sore throat (62.3%), and dyspnea (64.1%), while the features of COVID-19 patients with AKI were presented with cough (72.5%), fever (85.7%), sore throat (18.7%), and dyspnea (84.6%). In this cohort study, there is no statistical significance in age between COVID-19 patients with or without AKI (mean, 57.5 years versus 55.0 years, respectively). Additionally, patients who developed AKI had no any significant relationship with comorbid conditions including diabetes mellitus, heart disease, cancer, and hypertension. The AKI group had higher levels of inflammatory markers and kidney function tests (D-dimer, C-reactive protein, serum creatinine, blood urea) than the non-AKI group. On the other hand, the absolute number of patients needing hemodialysis was 34 (37%). This appeared in 5.2% of all patients and 37% of COVID-19 cases with AKI. COVID-19 AKI cases who needed hemodialysis had significant higher percentages with comorbid conditions including diabetes mellitus (73.5% vs. 14%), cardiac disease (23.5% vs. 5.2%), cancer (20.6% vs. 7%), and hypertension (79.4% vs. 28%). This group of patients had also the highest levels of inflammatory markers and kidney function tests [D-dimer (1495.5 vs. 1445.7 mg/ml), C-reactive protein (1017 vs. 626.5 mg/dl), serum creatinine (8.9 vs. 2.65 mg/dl), and blood urea (137.5 vs. 60 mg/dl)] in comparison to AKI patients who did not require hemodialysis. Another concerning observation was that 76% of the severe ICU-COVID-19 patients who developed AKI required hemodialysis, while 24.5% of the mild hospitalized COVID-19 patients who developed AKI required hemodialysis. Patients who developed AKI and were isolated at home did not require hemodialysis. The overall mortality rate in this study was 6.3% (41/651). The mortality rate was 15.4% (14/91) and 4.8% (27/560) in COVID-19 patients with or without AKI respectively. 38.3% of the patients who were required hemodialysis (13 out of 34) died during hospitalization, while 1.7% of the patients who did not require hemodialysis (1 out of 57) died during hospitalization. In COVID-19 patients with AKI who needed hemodialysis, significantly higher in hospital death rate was observed, as shown in Table 2.

**Table 1:** General characterization of study cohort

	No AKI patients (n=560)	AKI patients (n=91)	OR (95% CI)	***P. value***
**Variables**				
Age	55 (43–67)	57.5 (51–64)	6.3776 (4.1419–9.8202)	**< 0.001**
Male	320 (57.2%)	57 (62.6%)	1.0962 (0.7660–1.5687)	0.6157
BMI	29±1.5	30±2.6	6.3660 (3.6494–11.1050)	**< 0.001**
**Comorbidities**				
Diabetes	264 (47.14%)	33 (36.2%)	0.7692 (0.5032–1.1758)	0.2255
Hypertension	380 (67.8%)	53 (58%)	0.8583 (0.5971–1.2338)	0.4092
Cardiac disease	74.0 (13.2%)	11 (12%)	0.9148 (0.4676–1.7894)	0.7947
Asthma	31.0 (5.53%)	2 (2.0%)	0.3970 (0.0934–1.6875)	0.2108
Chronic liver disease	108 (19.3%)	6 (6.5%)	0.3419 (0.1459–0.8010)	**0.0135**
Cancer	28.0 (5.0%)	11 (12%)	2.4176 (1.1630–5.0256)	**0.0181**
**Presentations**				
Fever	304 (54.3%)	78 (85.7%)	1.5789 (1.1316–2.2031)	**0.0072**
Cough	512 (91.4%)	66 (72.5%	0.7933 (0.5653–1.1132)	0.1803
Dyspnea	359 (64.1%)	77 (84.6%)	1.3199 (0.9478–1.8381)	0.1004
Sore throat	349 (62.3%)	17 (18.7%)	0.2998 (0.1756–0.5117)	**< 0.001**
Diarrhea	86.0 (15.4%)	17 (18.7%)	1.2165 (0.6911–2.1412)	0.4970
Fatigue	251 (45.0%)	37 (40.6%)	0.9071 (0.6019–1.3671)	0.6414
Abdominal pain	39.0 (6.9%)	8 (8.8%)	1.2623 (0.5716–2.7879)	0.5644
Myalgia	143 (25.5%)	16 (17.5%)	0.6885 (0.3924–1.2080)	0.1932
**Laboratory results**				
HB%, g/dL	13.55 (12.3–14.8)	11.6 (10.0–13.2)	5.2747 (2.3648–11.7656)	**< 0.001**
WBCs, ×10^3^/μ L	7.7 (5.9–9.5)	10.0 (5.7–14.5)	7.6923 (2.9579–20.0045)	**< 0.001**
PLT, ×10^3^/μ L	222.5 (160–285)	173.4 (107–239.8)	4.8017 (3.5668–6.4640)	**< 0.001**
CRP, mg/dL	306 (64–600)	920.5 (566–1275)	18.5219 (14.3312–23.9380)	**< 0.001**
B. urea, mg/dL	32.5 (20–45)	135 (70–200)	25.1748 (16.2072–39.1044)	**< 0.001**
S. creatinine, mg/dL	0.9 (0.6–1.2)	7.5 (1.3–13.7)	43.0769 (5.2382–354.2467)	**< 0.001**
D-dimer, mg/mL	561.5 (233–890)	1435.75 (891.5–1980)	15.7241 (12.3437–20.0301)	**< 0.001**
**Type of isolation**				
Home	120 (18.4%)	13 (14.3%)	0.6667 (0.3609–1.2315)	0.1953
Hospital	327 (50.3%)	49 (53.8%)	0.9221 (0.6350–1.3392)	0.6702
ICU	204 (31.3%)	29 (31.9%)	0.8748 (0.5591–1.3687)	0.5581
**Outcomes**				
Cure	533 (95.2%)	77 (84.6%)	0.8890 (0.6418–1.2315)	0.4792
Death	27 (4.8%)	14 (15.4%)	3.1909 (1.6126–6.3140)	**< 0.001**

**Table 2:** Comparison between COVID-19 patients with AKI (yes/no—hemodialysis)

Variables	Hemodialysis yes n= 34/91 (37%)	Hemodialysis no n=57/91 (63%)	OR (95% CI)	***P. value***
**Comorbidities**				
Diabetes	25 (73.5%)	8 (14.0%)	5.2390 (2.1250–12.9162)	**< 0.001**
Hypertension	27 (79.4%)	16 (28.0%)	2.8290 (1.3361–5.9901)	**0.006**
Cardiac disease	8 (23.5%)	3 (5.2%)	4.4706 (1.1099–18.0070)	**0.0351**
Asthma	1 (2.9%)	1 (1.7%)	1.6765 (0.1015–27.6852)	0.7180
Liver disease	4 (11.7%)	2 (3.5%)	3.3529 (0.5828–19.2898)	0.1753
Cancer	7 (20.6%)	4 (7.0%)	2.9338 (0.7997–10.7633)	0.1046
**Presentations**				
Fever	32 (94.1%)	46 (80.7%)	1.1662 (0.6276–2.1672)	0.6267
Cough	30 (88.2%)	36 (82.5%)	1.3971 (0.7335–2.6610)	0.3091
Dyspnea	34 (100%)	33 (57.9%)	1.7273 (0.9106–3.2762)	0.0942
Sore throat	5 (14.7%)	12 (21.0%)	0.6985 (0.2265–2.1547)	0.5324
Diarrhea	7 (20.5%)	10 (17.5%)	1.1735 (0.4086–3.3709)	0.7663
Fatigue	25 (73.5%)	12 (21.0%)	3.4926 (1.5556–7.8419)	**0.0024**
Abdominal pain	6 (17.6%)	2 (3.5%)	5.0294 (0.9604–26.3386)	**0.0559**
Myalgia	8 (23.5%)	8 (14.0%)	1.6765 (0.5762–4.8781)	0.3430
**Laboratory results**				
HB%, g/dL	10.5 (10–11.0)	12.2 (11.2–13.2)	1.3971 (0.5454–3.5785)	0.4859
WBCs, ×10^3^/μ L	11.7 (8.9–14.5)	7.3 (5.7–8.8)	2.6345 (0.9326–7.4421)	0.0675
PLT, ×10^3^/μ L	224.9 (210–239.8)	164.5 (107–222)	2.2861 (1.4291–3.6571)	**0.006**
CRP, mg/dL	1017 (760–1275)	626.5 (566–687)	2.7193 (1.7579–4.2063)	**< 0.001**
B. urea, mg/dL	137.5 (75–200)	60 (46–74)	3.8279 (2.2713–6.4514)	**< 0.001**
S. creatinine, mg/dL	8.9 (4.1–13.7)	2.65 (1.3–4.0)	5.0294 (1.2730–19.8710)	0.0212
D-dimer, mg/mL	1495.5 (1011–1980)	1445.7 (891.5–2000)	1.7344 (1.1273–2.6685)	0.0122
**Type of isolation**				
Home	0.00	13 (22.8%)	0.0617 (0.0036–1.0715)	0.0558
Hospital	12 (35.3%)	37 (64.9%)	0.5437 (0.2499–1.1830)	0.1245
ICU	22 (64.7%)	7 (12.3%)	5.2689 (2.0363–13.6332)	**< 0.001**
**Outcomes**				
Cure	21 (61.7%)	56 (98.2%)	0.6287 (0.3258–1.2130)	0.1663
Death	13 (38.3%)	1 (1.8%)	21.7941 (2.7286–174.0770)	**0.0037**

## Discussion

In the current study, we assessed the frequency and severity of acute kidney injury in COVID-19 patients, as well as its impact on clinical outcomes. A total of 734 COVID-19 patients admitted to the Ahmad Maher Teaching Hospital in Cairo, Egypt, between June 6 and July 25, 2020, were examined. We excluded 83 patients on various levels of maintenance dialysis and renal transplant recipients. This study’s comprehensive analysis included data from 651 COVID-19 patients. The overall incidence rate of AKI among COVID-19 patients was 14% (91/651); AKI developed in 78 (14.7%) hospitalized patients and 13 (10.8%) home-isolated COVID-19 patients. Several previous studies found that the incidence of AKI in COVID-19 patients ranged from 0 to 36.6% [10–12]. When compared to SARS-CoV-1, SARS-CoV-2 appeared to have a higher frequency rate of AKI. An analysis of 536 SARS-1 patients revealed that 36 (6.7 %) of infected people developed acute kidney injury [13]. There were lower AKI occurrences ranging from 0 to 5.1% in the early studies of patients with COVID-19 [14, 15]. A systematic review of 24 reports on 4963 patients with COVID-19 found that the frequency rate of AKI was 4.5% [14, 16]. Our findings are significantly higher than those reported in SARS-1 or early COVID-19 patient reports. However, our findings are lower than those of a recent US cohort study, which estimated that 36.6% of patients progressed to AKI [7]. This disparity in the occurrence of AKI in patients with COVID-19 may be partly explained by the variable hospital admission conditions, the definition of AKI as well as the variance between demographic features, seriousness, riskiness factors, and outcome characteristics of the study’s populations. Current evidence indicates that AKI is an outcome of viral infection intermediate injury interactions, a dysfunction immune response, angiotensin II pathway activation, and hyper-coagulation, microangiopathy in patients with COVID-19 [2]. Other known risk factors for acute kidney injury, such as hypoxia that worsens over time and sepsis, are likely to interfere with COVID-19’s unique factors. We thought our incidence was higher than that of two Asian countries (0–9.2%) [4, 5]. However, it is lower than in more recent Italian and American reports (27.8% and 36.6%, respectively) [7, 17]. None of the reports include the inner city or lower socioeconomic communities with a majority minority population. However, we discovered a higher prevalence of comorbid conditions. When compared to a recent report, our study has about twice as many (66.5%) people with hypertension and more than three times as many (45.6%) people who are diabetic. Furthermore, compared to those who did not have AKI, the rates of ICU admission and in-hospital mortality were significantly higher. These findings were consistent with the outcomes of previous reports [17–19]. On the other hand, we assessed AKI patients’ recovery; 77 of 91 COVID-19 patients with AKI have recovered. When compared to the previous report conducted in China, our study has a recovery rate (84.6%) that is more than four times as high in China (18.2%). The rate of recovery in this study is higher than in a recent report conducted in Korea (67%). According to numerous studies, AKI is significantly associated with increased mortality [6, 20]. Several studies have documented that AKI has been related with a poor diagnosis among COVID-19 individuals [20]. In accordance with these reports, the current study found that COVID-19 patients with AKI have a threefold higher in-hospital mortality rate than COVID-19 patients who do not have AKI (15.4% vs. 4.8%, respectively). The total of AKI COVID-19 patients who required hemodialysis was 34. This accounted 5.2% of all COVID-19 patients and 37% of those with AKI. The mortality rate among this group of patients was 38.3% (13/34). In comparison to previous studies, the current study’s in-hospital mortality rate is lower than that reported by Hirsch et al. [7], who documented mortality rate of 34.8% in AKI and 5.6% in non-AKI. There are some limitations to this study. Due to a lack of medical services, we were unable to receive complete lab findings, such as urine analysis results. As a result, the impact of COVID-19 infection on urine cannot be described. Furthermore, we did not obtain the ventilator parameters, which may be important in lung-renal communications. As a result, the findings of our study required additional validation. This study was conducted during the COVID-19 pandemic in a single medical center in Cairo governorate; thus, we were unable to generalize our findings to all individuals infected with COVID-19 in Egypt.

## Conclusion

According to the findings of this study, AKI occurs in 14% of COVID-19 patients. Our findings suggest that COVID-19 cases, particularly those in the ICU, should be closely monitored for the progression of AKI. Early detection of AKI and prompt meditation may improve COVID-19 patients’ outcomes.

## Data Availability

The datasets used and/or analyzed during the present study are available from the corresponding author upon reasonable request.

## Abbreviations

ACE-2: Angiotensin enzyme-2
AKI: Acute kidney injuring
ARDS: Acute respiratory distress syndrome
CI: Confidence intervals
COVID-19: Coronavirus disease-2019
HD: Hemodialysis
ICU: Intensive care unite
Ors: Odds ratios
RT-PCR: Reverse transcriptase polymerase chain reaction
SARS-CoV-2: SARS-coronavirus-2

## References

[1] Guan W, Ni Z, Hu Y, Liang W, Ou C, He J, Liu L, Shan H, Lei CL, Hui DSC, du B, Li LJ, Zeng G, Yuen KY, Chen RC, Tang CL, Wang T, Chen PY, Xiang J, Li SY, Wang JL, Liang ZJ, Peng YX, Wei L, Liu Y, Hu YH, Peng P, Wang JM, Liu JY, Chen Z, Li G, Zheng ZJ, Qiu SQ, Luo J, Ye CJ, Zhu SY, Zhong NS, China Medical Treatment Expert Group for Covid-19 (2020). Clinical characteristics of coronavirus disease 2019 in China. N Engl J Med.

[2] Robbins-Juarez SY, Qian L, King KL, Stevens JS, Husain SA, Radhakrishnan J (2020). Pathophysiology of acute kidney injury in patients with COVID-19. BMC Pulm Med.

[3] Martinez-Rojas MA, Vega-Vega O, Bobadilla XNA (2020). Is the kidney a target of SARS-CoV-2?. Am J Physiol Ren Physiol.

[4] Cheng Y, Luo R, Wang K, Zhang M, Wang Z, Dong L, Li J, Yao Y, Ge S, Xu G (2020). Kidney disease is associated with in-hospital death of patients with COVID-19. Kidney Int.

[5] Wang L, Li X, Chen H, Yan S, Li D, Li Y, Gong Z (2020). Coronavirus disease 19 infection does not result in acute kidney injury: an analysis of 116 hospitalized patients from Wuhan, China. Am J Nephrol.

[6] Pei G, Zhang Z, Peng J, Liu L, Zhang C, Yu C, Ma Z, Huang Y, Liu W, Yao Y, Zeng R, Gang X (2020). Renal involvement and early prognosis in patients with COVID-19 pneumonia. J Am Soc Nephrol.

[7] Hirsch JS, Ng JH, Ross DW, Sharma P, Shah HH, Barnett RL, Hazzan AD, Fishbane S, Jhaveri KD, Abate M, Andrade HP, Barnett RL, Bellucci A, Bhaskaran MC, Corona AG, Chang BF, Finger M, Fishbane S, Gitman M, Halinski C, Hasan S, Hazzan AD, Hirsch JS, Hong S, Jhaveri KD, Khanin Y, Kuan A, Madireddy V, Malieckal D, Muzib A, Nair G, Nair VV, Ng JH, Parikh R, Ross DW, Sakhiya V, Sachdeva M, Schwarz R, Shah HH, Sharma P, Singhal PC, Uppal NN, Wanchoo R, Bessy Suyin Flores Chang, Ng JH (2020). Acute kidney injury in patients hospitalized with COVID-19. Kidney Int.

[8] Matuszkiewicz-Rowińska J, Małyszko J (2020). Acute kidney injury, its definition, and treatment in adults: guidelines and reality. Polish Arch Intern Med.

[9] Duff S, Murray PT (2020). Defining early recovery of acute kidney injury. CJASN.

[10] Naicker S, Yang CW, Hwang SJ, Liu BC, Chen JH, Jha V (2020). The novel coronavirus 2019 epidemic and kidneys. Kidney Int.

[11] Haibo Z, Penninger JM, Li Y, Zhong N, Slutsky AS (2020). Angiotensin-converting enzyme 2 (ACE2) as a SARS-CoV-2 receptor: molecular mechanisms and potential therapeutic target. Intensive Care Med.

[12] Pan XW, Xu D, Zhang H, Zhou W, Wang L-h, Cui X-g (2020). Identification of a potential mechanism of acute kidney injury during the COVID-19 outbreak: a study based on single-cell transcriptome analysis. Intensive Care Med.

[13] Zou X, Chen K, Zou J, Han P, Hao J, Han Z (2020). Single-cell RNA-seq data analysis on the receptor ACE2 expression reveals the potential risk of different human organs vulnerable to 2019-nCoV infection. Front Med.

[14] Takahashi T, Ellingson MK (2020). Sex differences in immune responses that underlie COVID-19 disease outcomes. Nature.

[15] Williamson EJ, Walker AJ (2020). Factors associated with COVID-19-related death using OpenSAFELY. Nature.

[16] Breif Communication (2020). Report on the epidemiological features of coronavirus disease 2019 (covid-19) outbreak in the republic of korea from January 19 to March 2, 2020. J Korean Med Sci.

[17] Vena A, Giacobbe DR, Di Biagio A, Mikulska M, Taramasso L, De Maria A (2020). Clinical characteristics, management and in-hospital mortality of patients with coronavirus disease 2019 in Genoa, Italy. Clin Microbiol Infect.

[18] Luo L, Fu M (2020). The potential association between common comorbidities and severity and mortality of coronavirus disease 2019: a pooled analysis. Clin Cardiol.

[19] Jafari A, Falahatkar S, YektaKooshali MH. COVID-19 clinical characteristics, complications and comorbidity: an updated systemic review and meta-analysis. 2020). Available at SSRN: https://ssrn.com/abstract=3631253 or. 10.2139/ssrn.3631253.

[20] Paek JH, Kim Y, Park WY, Jin K, Hyun M, Lee JY (2020). Severe acute kidney injury in COVID-19 patients is associated with in-hospital mortality. PLoS One.

